# “Navigating Risk and Responsibility?”: A Mixed-Methods Study Addressing Stigma and Well-Being Among Men Who Have ‘Sex on Chems’ with Other Men in the English Midlands

**DOI:** 10.3390/healthcare12232479

**Published:** 2024-12-08

**Authors:** Amanda Wilson, Iain R. Williamson

**Affiliations:** Psychology Division, Faculty of Health and Life Sciences, School of Applied Social Sciences, De Montfort University, Leicester LE1 9BH, UK; iwilliamson@dmu.ac.uk

**Keywords:** Chemsex, GBMSM, risk, responsibility, stigma, mental health, England, east midlands

## Abstract

Background: Most research on ‘Chemsex’ has been conducted with gay, bi-sexual, and men who have sex with men (GBMSM) in large cities with well-established infrastructures. Therefore, this study aimed to explore the ‘Chemsex’ risks and responsibilities of GBMSM who lived outside of the queer metropolis. This study also aimed to understand how stigma and mental health present in the absence of a well-established community infrastructure. Methods: This study utilized mixed methods in Leicester, Leicestershire, and Rutland, a location in the East Midlands of England. The quantitative component consisted of a survey. The qualitative component comprised three case studies based on in-depth interviews, using interpretative phenomenological analysis. Results: Of the 123 survey respondents, 86% engaged in riskier sex during sessions and 35% no longer enjoyed sober sex. In the interviews, the three men balanced risk management and personal responsibility, positioning themselves as mature, considerate, and well-educated users. All were alienated by the term ‘Chemsex’. The findings are considered using theoretical frameworks, or how discourses of ‘moral threat’ operate within the micro-politics of regulating GBMSM’s pleasure and stigma. Conclusions: The complexities of understanding this practice for researchers and practitioners are discussed and recommendations are made for reframing education and support services for GBMSM.

## 1. Introduction

### 1.1. Background

‘Chemsex’ is a term gay, bisexual, and other men who have sex with men (GBMSM) have constructed as the practice of using drugs with the intention of engaging in sexual practices, often with a group of men over a period of several days [1]. The terminology ‘Chemsex’ is both contested and contentious among researchers as well as GBMSM. A scoping review showed definitions are far from universal, with national and international definitions varying; however, what is agreed is that the practice involves having sex while under the influence of substances [2]. In his ground-breaking article, Stuart [3] discussed the London origins of the term ‘Chemsex’, assumed to apply nationally in the UK, which often combines ‘pleasurable’ drugs referred to in the community as ‘chems’, consumed alongside ‘functional’ drugs such as Viagra to maintain a sustained erection. This definition comes in part from allies and activists in the gay community as an effort to reduce stigma and allow for a term to emerge from a community, language which should be used by public health and governmental bodies that aims to address the risks associated with ‘sex on chems’. Stuart further argued how the term, whilst in some ways problematic, is needed for the provision of ‘culturally competent care’. Various drugs, or ‘chems’, commonly used in London- and Manchester-based ‘Chemsex’ include ‘Tina’ or crystal methamphetamine, ‘G’ or γ-hydroxybutyrate (GHB), γ-butyrolactone (GBL), and ‘Cat’ or mephedrone [4,5], and in Brighton, the substances include the aforementioned in addition to ketamine [6]. The substances consumed during sessions varies internationally as well, with a systematic review and meta-analysis in Europe concluding that there are different drugs used, but all studies agreed on the use of Tina, GHB/GBL, and mephedrone [7]. Indeed, in Asia, another systematic review and meta-analysis found the most common substances used during ‘Chemsex’ include the aforementioned list plus cocaine [8]. Drugs are consumed in various ways including orally, being smoked, or being injected intravenously (known as ’Slamsex’ in UK). Motives for engaging in ‘Chemsex’ appear to be multiple and complex [9].

### 1.2. Literature

Most research in this area comes from a public health perspective seeking to understand the motives to engage in ‘Chemsex’ and to reduce associated risks to physical and psychological well-being [10]. Engaging in ‘Chemsex’ increases the risk of sexually transmitted infections (STIs) [11], injecting problems (abscesses, ulcers, endocarditis, deep vein thrombosis, etc.) [12], and overdose/overdose-related deaths [13]. Higher rates of STIs have been reported by those engaging in ‘Chemsex’, with strong associations reported between the acquisition of various STIs, including the human immunodeficiency virus (HIV) and Hepatitis C, due to riskier sexually behaviours [14]. GBMSM who engage in ‘Slamsex’ are at an increased risk of contracting blood-borne viruses (BBVs) [15,16], with the late diagnosis of HIV among injecting drug users increasing across the United Kingdom [17]. The widespread use of pre-exposure prophylaxis (PrEP) in the GBMSM community offers protection against HIV transmission but not other STIs, and there may be challenges to PrEP adherence, especially during extended ‘Chemsex’ sessions [18]. In a study by Pufall and colleagues [19] on HIV positive men engaging in ‘Slamsex,’ the men reported adhering to Anti-Retroviral Therapies (ART), reducing the risk of HIV transmission when there are no drug interactions. Even when ART, condoms, and/or PrEP are used to protect HIV-negative partners from transmission, there are other health concerns such as the higher risk of penile abrasions and/or rectal tears, which are related to the amount of time spent engaged in a session [20]. Furthermore, cognitive capacity to give consent to sex has been a harm-reduction concern specific to ‘Chemsex’, with the need to promote paying attention to sexual partner(s) capacity being highlighted alongside increased education being delivered on the risks associated with psychoactive substances [21]. As Morris [22] argues, in ‘littoral spaces’ where fantasy and reality combine, consent dynamics become more complex, putting some men at elevated risk of rape, other forms of sexual assault, and being ‘stealthed’ (wrongly believing that a sexual partner is using a condom during anal sex). The rates of STIs, BBVs, physical health concerns, and mental health disorders like anxiety or depression have been reported to be high among those engaging in ‘Chemsex’ [19], suggesting a need for models of care and support that take an integrated and holistic approach to psychological and physical health in this population. Two services, drug harm reduction and GBMSM sexual health clinics, currently serve those engaging in ‘Chemsex’, making them ideally placed as a starting point for an integrated holistic model.

Harm reduction advice often centres on educating users on the drug metabolic pathways, routes of administration, bioavailability, pharmacokinetics, half-life, the risks of poly drug use, and possible interactions with HIV medicines [23]. GBMSM are presenting at sexual health clinics (SHCs) across England, instead of drug services where harm reduction workers are placed, and SHC workers are conducting ‘Chemsex’ consultations on a monthly basis, emphasising that there is a need for both specialist harm reduction training and specialist ‘Chemsex’ services in order to reduce the health issues individuals are presenting with [24]. Within both services, there is a need for understanding local community contexts as the ‘Chemsex’ scene lacks homogeneity, with categorisation occurring based on the drugs used, the frequency of use, the locale and intensity of use, the mode of administration, and HIV status [25]. Service-users’ accounts of their experiences across England are mixed, with many GBMSM describing satisfaction with service providers but others citing significant under-resourcing, challenges in accessing appropriate services, and, in some cases, perceived stigmatising attitudes [26]. It is apparent that there is a need for culturally/locally sensitive and effective health promotion that employs an appropriate harm-reduction framework, accepting that some people will want and continue to have ‘Chemsex’ despite awareness of the risks it poses [27]. As a risk factor for several serious health and psychological conditions, it is important to ensure integrative and holistic services are provided for this marginalized community.

While most public health literature treats ‘Chemsex’ as a concern, there is research globally that suggests ‘Chemsex’ can be responsible and pleasurable. In Aotearoa, New Zealand, and Australia, interviews with 16 gay men suggest Chemsex as an expression of wild self-care, or where practices deemed dangerous are viewed as life affirming and liberating [28]. In Quebec, Canada, pleasure in methamphetamine use has also been reported, with more acceptance, higher performance, and increased sexual exploration during Chemsex parties [29]. In a more similar European context, research from France has identified the complexities of pleasure during Chemsex, such as connection, the disinhibition of internalized homophobia, and as stress relief [30]. Given that research in the United Kingdom is often conducted on those who have a substance-abuse issue who have engaged in ‘Chemsex’, it is important to understand that dimensions of pleasure should be considered, with its complexities, while at the same time recognising and understanding elements of risk.

### 1.3. Rationale, Aims, and Research Questions

British research on ‘Chemsex’ has overwhelmingly been conducted on GBMSM communities in large cities such as London, Brighton, and Manchester, which have significant visible GBMSM communities and service infrastructure [5,11,31]. However, ‘Chemsex’ occurs away from the ‘Queer Metropolis’ [32,33] and little is known about the community of GBMSM who have ‘Chemsex’ outside these main cities. British researchers have thus far arguably been less agile in contextualising ‘Chemsex’ away from major urban centres and white voices have been over-represented. Therefore, the present study focused on the Midlands of Leicester, Leicestershire, and Rutland (LLR), a culturally diverse shire but a relatively impoverished city of around 368,600 residents. There are two local charities providing services for GBMSM, though they serve the wider lesbian, gay, bisexual, and transgendered (LGBT+) community in LLR. One of these charities specializes in sexual health and further runs a drop-in clinic with the local drug services specifically for ‘Chemsex’; although, you need to be able to access the charity, which can be difficult for residents of the rural boroughs that compose LLR. The local government, as part of their Sexual Health Strategy from 2020 to 2023, aimed to strengthen links between sexual health, drug services, and health promotion, in particular for MSM, when providing Chemsex services [34]. However, the local government have yet to evaluate their 2020–2023 strategy in reaching the target of strengthening links for ‘Chemsex services’, nor have they published their 2024–2027 strategy, and the LGBT+ charities locally were not listed as part of the face-to-face consultations to formulate the next local government strategy [35]. This is likely a result of public sector cuts that have been impacting the provision of local government services in England since 2007, resulting in a lack of nationally protected standards of minimum care for this minority community [36], with funding cuts being disproportionate for the LGBT+ community [37]. LLR has, as a result, lost its only HIV support-focused charity, which supported recruitment to this study. This reinforces again the differences in provision across England. The ‘queer metropolis’, with its built-in infrastructure, however, shows less impact on LGBT+ government service provision, for example, Brighton City Council has funded published research on the effectiveness of HIV and STI test vending machines [38]. Given the wide variation of the impact of funding cuts on local authorities and the lack of evaluation of the local sexual health strategy, it was important to conduct this study to better understand the needs of those engaging in ‘Chemsex’ in LLR. Furthermore, LLR has one of the highest migrant populations outside of London, with the most recent census in 2021 reporting that 42.1% of LLR residents were born outside of England, with the majority being born in India, South and Eastern Africa, Poland, and Kenya [39]. While little is known about ‘Chemsex’ in India, research suggests only 5% of MSM engage in ‘Chemsex’ and more use alcohol (23%), while other Asian countries report higher prevalence rates, due to cultural aspects like less accessibility to drugs and more stigma around drugs and homosexuality [40]. In Poland, amyl nitrate, cannabis, alcohol, and psilocybin are the dominant drugs used during ‘Chemsex’, and these are seen as a coping mechanism for stress [41]. There is no research on ‘Chemsex’ and Indian migrants, Africa or African migrants, Polish migrants, and Kenya or Kenyan migrants, leaving a gap in how to provide culturally competent support to these communities in LLR, nor is there information on ‘Chemsex’ available in these countries’ native languages.

Utilising a mixed-methods approach, this study begins to close the gap in British research on the attitudes and experiences of GBMSM based away from the queer metropolis who report engaging in ‘Chemsex’. The design allows for a localised understanding of ‘Chemsex’ as a social–sexual phenomena to emerge and inform our understanding of GBMSM health and well-being. Therefore, this study aimed to explore attitudes and reported behaviors around ‘Chemsex’ of GBMSM in the East Midlands of England. The study also aimed to understand how stigma and mental health manifest and are managed in the absence of a well-established community infrastructure.

The research questions were as follows:

What are the reported ‘Chemsex’ behaviours of men in Leicester, Leicestershire, and Rutland? (Phase 1—Quantitative)

How do ‘Chemsex’ participants in these areas describe and make meaningful their involvement in these behaviours? (Phase 2—Qualitative)

How do GBMSM manage the experience of actual or anticipated risks to health and well-being during ‘Chemsex’? (Integration of Phase 1 and Phase 2 findings)

## 2. Materials and Methods

### 2.1. Design

This study used a two-phase mixed-methods approach which aligned with an Explanatory Sequential Design [42]. The initial quantitative part comprised a community survey and this was followed up by a series of in-depth individual interviews with regular ‘Chemsex’ participants to further contextualize the phenomena. First, the quantitative data were collected and analysed; this was performed to scope the substances used, as well as to gather attitudes on behaviors using a larger sample that was more reliable. The results from the first phase were then used to compose the interview topic schedule for the second phase. Substances used were collected in the survey and a demographic slip was collected during the interviews to triangulate the substances used, and the interview topic schedule then further explored the behaviors and attitudes through participants’ lived experiences to garner rich details of the local ‘Chemsex’ scene. Once the qualitative interviews were conducted and analysed, then the findings from both phases were integrated and interpreted in the discussion to understand how GBMSM managed their health, well-being, and any risks, as well as what converged and diverged in the data sets. Ethical approval for this study was provided by the appropriate Faculty Ethics Committee at the authors’ University, following the Declaration of Helsinki, revised 2013. A research proposal was created to act like a protocol for the project, which was reviewed by the Ethics Committee prior to the study commencing, with attention paid to legality due to the participants using illicit substances. Informed consent was gathered, ensuring all details required about the project were in the participant information sheet, with time given to discuss the information sheet and have any questions answered. No participant could be under the influence of drugs during either phase of the study to avoid questions being raised around ability to consent to participation.

### 2.2. Quantitative Component

An online pilot survey design with multiple recruitment strategies was advertised and made available to participants via various fora between 22 August 2017 and 31 January 2018. The survey was designed by the local LGBT+ sexual health service, due to their local intelligence on the ‘Chemsex’ scene from their support clinic. At the time our pilot survey was conducted there was no validated measure for ‘Chemsex’ in the British cultural context to utilize [43], though our survey questions capture similar data to other published self-constructed surveys around this time [5,11]. This research study was promoted at Leicester Pride in September 2017 through social media and weblinks on Leicester-based sexual health (Trade Sexual Health) and substance use (Turning Point) support agencies as well as via geospatial dating/hook-up apps commonly used by GBMSM men (Grindr and Scruff). Men who were currently using recreational drugs in sexualised settings or who had done so in the past and spoke English were asked to participate. To promote inclusivity and accessibility, the survey took no longer than ten minutes to complete, and after a short demographics section, included a series of items around substances used, concerns around safety, and psychological aspects of ‘Chemsex’ behaviour and ongoing ability to enjoy ‘sober sex’ (i.e., sex with substances) using a standard five-point Likert scale of agreement. An open-ended item asked about preferred contexts for information and support.

### 2.3. Participants

One hundred and twenty-three people completed the survey in full. It is difficult to conclude if the sample is representative for several reasons relating to rigorous data collection. The Leicester Health and Wellbeing Survey 2018 suggested around 4% of residents (around 13,200) 16+ identified as LGB [44]. However, in 2021, the Office for National Statistic (ONS) stated data on sexual orientation from local authorities is generally regarded to be relatively unreliable, rationalizing the first census on sexual orientation [45]. The ONS 2021 census suggested 3.1% (11,439 residents) identified as LGB [46]. Both the 2018 and 2021 data set population estimates should be taken with caution. They fail to separate the responses of Gay and Lesbian residents, to provide a way to capture men who have sex with men, and it is impossible to determine male from female bisexual residents, making any figure of GBMSM in LLR an overestimate. Regardless of the ability to ascertain if our sample was representative, results showed 72 (58%) reported that they had not taken recreational drugs in a sexual context within the last twelve months whilst 51 (42%) said they had. Of these 51 participants, 71% identified as gay, 25% as bisexual, and 2% as a heterosexual man who has sex with men. In total, 98% identified as cisgender and 2% as trans*, with 88% identifying as male and 12% as non-binary. It is important to note that non-binary people do not identify with the male gender, and that this is the first study outside the queer metropolis cities in the UK to highlight that non-binary people engage in ‘Chemsex’, suggesting a further need to look at this special group in future localized contexts. With regard to ethnicity, 68% were white British or white European, 20% were British Asian, 4% were Mixed Race, and 2% were Black. An amount of 51% lived in the City of Leicester, 45% lived in Leicestershire, whilst 4% lived in the small, largely rural, neighbouring county of Rutland. In total, 24% were below the age of 26 years, 66% were aged between 26 and 49, and 10% were over the age of 50. A small number of respondents chose ‘prefer not to disclose’ items on certain items, which explains where totals do not reach 100%. Items on HIV status or other aspects of reported sexual behaviour (such as condom or PrEP use) were not included in the survey.

### 2.4. Procedure

The survey used the Survey Monkey platform and was accessed via a web-link in the advertisements and social media posts. To access the GBMSM community, the researchers used multiple methods to advertise the study. Completion through the Trade Sexual Health website received the most responses followed by promotion of the study at Leicester Pride and subsequent social media promotion (64 respondents). Forty-two participants responded through Grindr advertisements (42 respondents) and thirteen through Scruff advertisements (13 respondents). Four responses came via miscellaneous other sources.

Ethical aspects followed published guidelines for internet-mediated research [47]. The survey had a short explanatory introduction and debriefing section for when participants completed or withdrew from the survey. All data were anonymous and no financial incentives were offered. Links to information and support services for those concerned about aspects of their behaviour, particularly in relation to sexual activity and/or substance use, were made available to all respondents.

### 2.5. Analysis

All findings are presented as descriptive data with frequencies and/or percentages.

### 2.6. Qualitative Component

In October 2018, the follow-up qualitative component was launched, which invited men who identified as GBMSM, over the age of 18 years, who were currently engaging in ‘Chemsex’ or had done so in the past twelve months to participate in individual interviews about their experiences. This study was advertised through social media and websites for Trade Sexual Health and Leicestershire AIDS Support Services (LASS). Because of significant recruitment difficulties, this study was re-promoted on two separate occasions in February and June 2019. Interviews utilized a semi-structured schedule and data have been analysed employing Interpretative Phenomenological Analysis (IPA) [48].

### 2.7. Participants

A convenience sample of three participants, who were all regular ‘Chemsex’ participants at the time of data collection, each took part in one-to-one interviews lasting between 60 and 75 min. The interviews were carried out in confidential LGBTQ-community spaces at Trade (N = 1), Leicester AIDS Support Service (N = 1), and a private room at the University (N = 1) in Leicester in 2019. Three other potential participants approached the research team and were sent the Participant Information Sheet but subsequently decided not to take part in this study.

The three participants were

Aabid, a gay HIV-negative British Indian man, aged 33, living in City of Leicester

Angus, a gay HIV-positive white Scottish man, aged 51, living in City of Leicester

Azaan, a gay HIV-negative British Pakistani man, aged 29, living in City of Leicester

### 2.8. Procedure

This section was guided by the British Psychological Society’s Ethics for Human Participants guidelines in terms of researcher competency (specialized knowledge of methods and topics), informed valid consent, not approaching participants directly to avoid coercion in participation, anonymity and confidentiality, and right of withdrawal, and a risk assessment was conducted due to the sensitive nature of the research [49]. Interviews were guided flexibly by a topic schedule of open-ended questions in part informed by the survey responses and reviewed by the recruiting charities to ensure they were appropriate and unlikely to cause distress. All men received an information sheet ahead of agreeing to participate, signed consent forms on the day of the in-person interviews, and received full debriefing with information about services and support. Support was available should anyone become distressed during the interviews by a health promotion worker, and the further resources included the local sexual health LGBT+ charity and the HIV charity. Additional online support was also provided signposting participants to the local drug services and online information from 56 Dean Street (a charity in London that was the first to provide GBMSM with online information regarding ‘Chemsex’). No participant became distressed during the interview process. Participants in phase 2 received a GBP20 voucher for a major online retailer as a thank-you and were also provided with a harm reduction ‘safer injecting’ kit by The Gay Men’s Health Collective which has a booklet specific to GBMSM for safer ‘Chemsex’ and includes measures to prevent blood-borne viruses, such as clean syringes and using condoms (see https://www.exchangesupplies.org/shopdisp_pip_pac_safer_injecting_and_safer_sex_pack.php (accessed on 27 October 2021)). Interviews were audio-recorded and transcribed in full using basic annotations to indicate pauses, emphasis, and use of louder or softer speech as well as key paralinguistic elements. All participants were provided with a pseudonym in the transcripts to protect their confidentiality and anonymity, with names and places that might identify the participants being removed or changed.

### 2.9. Analysis

Analysis followed the principles and procedures of IPA [45,50]. In particular, in this analysis, the researchers have foregrounded the idiographic sensibility of IPA and because of small sample size, the diversity of our participants and the depth of data elicited from each individual, we have presented the accounts as three case studies using a protocol outlined by Smith, Flowers, and Larkin [45]. Following data familiarisation and coding, a set of themes was produced for each participant and the most salient have been selected for presentation. Interpretative phenomenological analysis is especially well suited to in-depth case study data because of its theoretical ‘touchstones’ of idiography (retaining unique individual aspects of the cases discussed) and hermeneutics (whereby the analysis aims to represents a persuasive interpretation of each participant’s account co-constructed through both parties’ meaning-making processes).

## 3. Results

### 3.1. Phase 1—Quantitative

In this section, we present information about the substances used by the 51 men from the community study.

### 3.2. Reported Substances Used

In relation to substances used by respondents in the prior twelve months, cocaine and GHB/GBL were the most commonly used with a figure of 59% of participants (N = 30). These substances were followed by mephedrone (N = 22; 43% of respondents) and crystal methamphetamine (N = 19; 37%). The only other substance to be reported by a significant number of participants was ketamine (N = 14; 27%).

### 3.3. Attitudes and Reported Behaviours About Sexualised Substance Use

Participants were asked to rate seven items on a five-point Likert scale from strongly agree to strongly disagree. Responses are reported as percentages for each of these in Table 1.

The results show that a majority reported a fair degree of self-efficacy in safe drug-use and felt they knew where to get advice about drug-use. However, most also report engaging in sexual acts under the influence of drugs that they would not perform ‘sober’. Two-thirds continued to enjoy sober sex and the views on the extent to which participants felt that drugs had a negative effect on their lives were more evenly split than other items. However, 47% did indicate there was a negative effect, making it important to explore this item further in phase 2, both due to the more even split and the marginally stronger likelihood to agree on their negative impacts.

### 3.4. Preferred Source of Support and Information

One final question asked participants to indicate what sort of service they would feel most comfortable accessing advice about their drug-use. Responses here were rather mixed, with 39% preferring an LGBT service-provider; 21% electing for a drugs support agency; 20% said they would visit their GP; while 12% would access a specialist sexual health service. The remaining 8% identified a variety of other sources including looking online or asking friends and peers.

### 3.5. Phase 2—Qualitative Findings

#### 3.5.1. Case Study 1: Aabid: ‘Filling a Void’

Aabid was British Indian, identified as gay, practiced Islam, and was 33 years old. He described himself as a ‘*novice’* when it came to sexualised drug use, participating 10 times in different sessions and using more widely used drugs like cannabis and ecstasy. He reported maintaining good sexual health practices, such as testing for STIs/HIV every 3 months, and negotiating on Grindr the types of sexual encounter he wanted to have. For Aabid, ‘Chemsex’ meant ‘*organising to meet up with someone, and then that involves sex and drugs*’ but he noted that ‘*sex and drugs*’ was the term he preferred to use. He had been in relationships with other men in the past and had used ‘*sex and drugs*’ both with his former partner and other men, and then as a single man. For Aabid, ‘*sex and drugs’* was described as ‘*this feeling that you don’t feel at any other time’*, he engaged in it ‘*to have a really good time’*.

Aabid acknowledged the stigma that existed between his faith and his sexual orientation, which mostly occurred when he was younger and influenced his decision to start engaging in sex with drugs. He had sought support for his poor mental health while resolving his gay identity with his Muslim identity, but he felt this identify conflict was not his primary motivation for sex on drugs, ‘*I think it played some role I don’t know how big that part is*’. This uncertainty could be explained by the social support he was able to find within the more open part of his cultural community to buffer against any negative cultural stigma related to being Asian, Muslim, and attracted to the same sex,

*‘I come from a South Asian backgrounds erm you know there there’s lots of like rejection and lots of family problems and err because I come from a Muslim family there’s all these issues around that as well, often you want to feel accepted and you know often there are people, nice people, who are looking to make you feel accepted and therefore you turn to them’*

Stigma was indicated in his use of how he faced “lots of like rejection and lots of family problems” coming from a South Asian Muslim family. Despite this though, he had found ‘*nice people’* who made him ‘*feel accepted’* and therefore he felt he had social support to ‘*turn to’* as a gay Muslim man.

When asked further about the other factors, his narrative talked about a range of different aspects (including both social and existential components) behind why ‘*a novice’* would begin to engage in ‘*sex and drugs*’.

*‘Yeah, yeah or trying to feel better or trying to err, like err, fill a void or escape from reality or trying to be in a different situation or a different place or just trying something new for the first time erm, there’s lots of different things that come in to it like peer pressure and things like that I suppose as well.’*

The complex reasons behind ‘*sex and drugs*’ included to ‘*fill a void*’ and ‘*peer pressure*’, with ‘*peer pressure’* being something he thought might play a part but was unsure (*‘I suppose’*) of the influence. He explained further that filling a void was to ‘*escape from reality*’, suggesting it was to decrease stress rather than a maladaptive coping strategy.

For Aabid, it was important to have a sense of secure identity and confidence in himself so he could have a good time that was ‘*empowering*’ and he could be assertive. He had attended an assertiveness workshop, run by a charity in London on how to be assertive as a GBMSM when negotiating sexual practices, where he learned ‘*life skills’* that were ‘*quite useful’* and good to ‘*meet other people’*. Without these skills, he would not feel comfortable saying no:

*‘I think I’m getting better at it like the assertiveness where I’m trying to look after myself more and saying ‘no’ more more often and you know standing up for myself as it were so yeah I’m doing that more often. And it’s nice and it’s really empowering but er most of the exp most experiences you know having sex is is a positive one because it feels good.’*

He repeatedly used the word ‘*more*’ to indicate that he is becoming assertive and ‘*standing up for*’ himself as he ages. Having these skills made him feel ‘*positive*’ about having sex with drugs ‘*because it feels good*’. Risks emerged despite the skills Aabid developed, and he was unsure how to manage challenging ‘Chemsex’ scenarios. For example, he disclosed that if someone were to overdose, Aabid’s response would be to run away as he believed that he would receive a ‘*caution*’ if he called an ambulance, and said he would ‘*get scared*’. He was scared of his gay identity being exposed, implying a level of internalized stigma. While he mostly reported negotiating condom use, he did mention that there were times when you just did not use one, which ‘*depends on the other person*’. He also described a subsequent sense of ‘*panic’* both during HIV testing and during the waiting period before getting the results. In the past, he was treated for an STI and also had a fear of contracting HIV, increasing his panic. If his sexual partner disclosed that they were positive, he said he would ‘*just block em or if they tell me then I just sort of walk away*’, and this showed some stigma towards those living with HIV. These elements provide further evidence for Aabid’s ‘novice’ label and his recognition of this.

Another adversity Aabid faced was the small gay community and perceived enmeshed nature of Leicester’s Muslim community, which made him ‘*reluctant*’ to get support from the GP for ‘*sex and drugs*’, as the receptionist could be someone who lives on his street or knows his family. There were also concerns that the reason for his appointment would not be kept confidential and others in his community may find out sexual identity, which may be unknown to family, friends, others, etc., and could result in psychological or physical harm to an individual. Although Aabid refrained from using the term *Islamophobia* or *racism*, he did describe negative experiences such as *all that bitchiness and nastiness and being horrible* on the gay scene. Actual and felt stigma do appear to be a running theme in much of Aabid’s account—especially his challenges in integrating Muslim and gay identities—and self-stigma is shown in his fear of being exposed.

Have positive sexual and romantic experiences in a relatively small gay community like Leicester city seemed to be the void Aabid wanted to fill. Although becoming fairly comfortable with his gay identity, Aabid feared being exposed in various settings and whilst he appeared to have managed his own health and psychological risks effectively, he was ill-prepared for possible scenarios that might result during or after a ‘Chemsex’ session as a self-proclaimed novice.

#### 3.5.2. Case Study 2: Angus: ‘A Principled Approach to Play’

Angus was white and in his fifties. He described himself as very sexually experienced including several years as sex worker. He appeared very knowledgeable about the drugs used in ‘Chemsex’ sessions and how to manage risks. Angus was the most activist and politicised of the three men who were interviewed. Like other participants, he disliked the term ‘Chemsex’, suggesting it was a stigmatized word, calling it ‘*Daily Mail fodder… stigmatising by virtue of its name’.* He articulated how the use of substances to accompany sexual activity had a long history across contexts and cultures. In doing so, he repositioned ‘Chemsex’ as an extension of normative sexual practices, rather than a contemporary public health crisis associated with ‘*dirty depraved gay men’:*

*‘People have always used stimulants for sex, so... a joint, the gin and tonic generation, the Bacardi Breezer generation to… ecstasy and ketamine in the nineties…to drugs particular to a new wave or new generation of Chemsex… crystal meth, mephedrone or GLB GHB.’*

Key to understanding Angus’ approach to engaging in ‘Chemsex’ was a reported sense of protective respect for his own physical and psychological well-being and, as far as he was able, that of his partners and the parallel processes of managing risk and heightening enjoyment, which he saw as largely complementary. He described having a number of ‘*boundaries*’ stating he ‘*hadn’t gone to the extremities of Chemsex*.’ He enjoyed sessions relatively regularly but enjoyed sober sex and he kept clear parameters round his frequency of participation,

*‘It’s a place I choose to visit, not where I’d like to live. The Chemsex thing, it’s like a holiday destination... don’t pitch a tent!’*

He described himself as ‘*mindful of the risks’* and also carefully regulated the maximum time he would consider participating in a session (24 h)—arguing ‘*knowing when is enough is a lovely thing to be aware of’.* This appeared to be related to careful research, a small number of negative prior experiences (including one experience of being ‘double-dosed’), and observing other men overdosing and/or becoming very distressed. In managing those elements in a carefully organised, almost scripted, manner, Angus felt he created a rewarding experience for him and his sexual partners as a transformative dyadic act:

*‘I’ve always thought going into your head is much interesting than getting out of it… Having a heightened frame using stimulants and being heightened and present and unconditionally sharing yourself and the experience with somebody can be phenomenal… really kind of intense kind of heightened bonding… you take off at the same time and you go on a journey essentially together’*

Angus appeared to understand drugs and dosing well. He adopted a very disciplined ‘ritualised’ approach to ‘Chemsex’ informed by what he called the knowledge of ‘*First Nation persons’* around using psychoactive substances,

*‘It should be seen within ritual as I think really good sex should be. You’re not meant to live in the high, you’re not meant to live in the preparation [or] in the integration. They are just three key parts of the journey… You should honour all three parts of the processes with equal weight…That awareness for me was incredibly useful. It framed my engagement with Chemsex.’*

Angus outlined his processes to some length. Key for him was to ensure that he was taking part in ‘Chemsex’ when his body was ‘*cleansed’* and nourished, describing a regime of hydration and nutrition not dissimilar to an athlete before a sporting event, and including foods believed to boost serotonin that could bolster against poor mental health,

*‘Generally, I would always like to have some chicken or salmon fillets just the amount for protein, some potassium bananas, some fruit, some yoghourt and lots and lots of fluids.’*

Equally important was engaging in ‘Chemsex’ only when he felt psychologically ‘*resilient’* to buffer against the impact that the substances used could have on mental health*,*

*‘I make sure there is a grounded-ness... If you’re in a downer or if you’re feeling blue… or shit… it’s not a good thing to add further exhaustion to that. I don’t just mean physically, I mean emotional and spiritual exhaustion.’*

Similar attention to detail was applied to his partner before substances were consumed where they discussed ‘*consent around risk and around what’s permissible’*. He viewed the ongoing confirmation of mutual consent as a ‘*live document throughout’* the session.

To facilitate effective comedown, Angus pre-prepared post-session food and talked about learning how to ‘*integrat[e] your high into your everyday life.’* Within his approach, Angus recognised some of the risks he was not wholly able to control, he felt he was able to create a relatively safe environment through the application of and adherence to a clear set of principles and processes that he appeared to apply consistently. In doing so, he differentiated himself for many other (typically younger) men who he described as ‘*running faster than the speed of loneliness*,’ who he believed, partly through a lack of effective education, easily got caught into dangerous cycles of increasing drug strength and/or dosage, perceiving them as becoming ‘*stuck chasing the dragon or searching for that high, buying more drugs and trying to keep the party running.’* Rather than following a similar path, Angus argued that he was happy to ‘*revisit things that I have done well or experienced as being fruitful.’*

As an assertive, experienced gay man, Angus appears to exhibit very little sense of stigma relating to his identity or his sexualised drug-use. Essentially, he normalised the latter by explaining ‘Chemsex’ as one manifestation of using chemical substances in sexual enjoyment that transcends time, sexual communities, and cultures and that can be managed safely with appropriate knowledge and care.

#### 3.5.3. Case Study 3: Azaan: ‘A Marriage Made in Heaven’

Azaan was Pakistani Muslim, age 29, identified as gay, and did not engage in long-term relationships due to cultural stigma and the need to conceal his gay identity. Instead, he primarily purchased sex from escorts, and this division of his identity he reported as working for his lifestyle. He had engaged in ‘Chemsex’, or as he referred to it, ‘sex on chems’, over 100 times, mostly at weekends after a busy week’s work. He typically used methamphetamine and gamma hydroxybutyrate in combination,

*‘So err it gives you erm a happy feeling euphoria feeling erm […] and it gives you erm, it gives you extra err energy, endurance. It’s not like erm, so you, you could have like, I could have the longest day at work and I’ve not slept and physically my eyes are rolling but I have that and I’d be fully, I could do a ((stutters)) do another twenty four hours you know […] but it goes very well with G I find ah what I say its likes a marriage made in heaven err G and erm methamphetamine, whereas like cocaine alcohol those are like the two you know they tend to go well with each other.’*

When participating in the use of ‘G’ and ‘methamphetamine’, Azaan felt good, he felt *‘happy’*, *‘euphoria’*, *‘energy’*, *and ‘endurance’*, like he could ‘*do another twenty four hours’* of work. The extract presents the duality found within the participant’s feelings towards ‘*sex on chems’*, as the two drugs were described as ‘*a marriage made in heaven’*. This metaphor used by this participant to describe the duality of control and release was frequently present within his interview, for example, he discussed the use of cocaine and alcohol, that it ‘*softens you that little bit’*, ‘*goes well together’*.

Despite the risk of polysubstance use: cocaine and alcohol, G and methamphetamine, Azaan’s talk suggested that he was relatively well educated on safer sex and drug-taking practices. He used condoms and this was negotiated before sex with the escort. He also used PrEP regularly, to maintain positive sexual and psychological health, as he would not need to worry. He engaged in STI testing every six months. Other safe practices included not putting G into anything plastic as it is a chemical that could ‘*react*’, so he stored it in a ‘*glass bottle’* and kept it out of the ‘*sunlight etc… a cool area’*. Furthermore, he labelled the container ‘*dangerous substance’* in case anyone was to find it and kept it out of easy reach of a child. Due to the chemical nature, he explained that you had to mix G with another liquid; he used a one millilitre syringe to measure his dose of G, to not fall asleep or overdose, and he would not ‘*take anything more than 3 mils within one hour* ‘*cause it could be fatal*.’ Azaan discussed that the strength of ‘Tina’ (methamphetamine) could be identified by appearance, ‘*the cleaner, the clear the crystal then you do know that it’s… more pure more clean*’, which determined how much he would take. Another way to test the purity was heating it up,

*‘when you administrate it, so when I like if I was to smoke it, erm when you’re heating it you get to notice the viscosity of the liquid… If it’s really runny (guess) then it’s a problem erm whereas if it’s a more thicker base and doesn’t burn so easily then you know you’ve got a good one.’*

Azaan used a glass fishbowl pipe to smoke, which again is generally regarded as good practice. He used in both Leicester, Leicestershire, Rutland and London and said that he saw far more people abusing the drugs and being dependent on G in Leicester (shire). This did suggest stigma towards those with substance-use disorders who engage in ‘sex on chems’ outside places with GBMSM infrastructure, where they were seen more in the image of a stereotypical drug abuser, as substance-use disorders do not discriminate based on location. He explained this dependency was due the purity of the drugs being worse outside London,

*‘That’s what happens in Leicester a lot, that’s what I find erm and then obviously you’ve got case I’ve come across a couple of people where they’ve become heavily dependent on it, they can’t, they need  G regardless.’*

The lack of quality control in Leicester was a primary reason he preferred to go to London, as well as there being more escorts.

Despite the perceived poor drugs quality, that *‘marriage made in heaven’*, of control and release, was why Azaan chose to engage in ‘sex on chems’, to bring that duality in his life, and to balance his mind,

*‘So it’s it’s totally a different life, so I get to see that, so that it makes me feel positive in that way erm but then when I am coming off it I’m thinking like ohh I’m just like ohh ((laughs))[…] I’m thinking like ohh I wish I could’ve (unclear) like that and then you hit reality and then you come back to that life but I’m glad I’ve got this in a way as well cause it’s keeping in more control if I want. If I never had it I’d be probably off the rails, I’d probably be I don’t know where I’d be at the moment.*

### 3.6. Interviewer: Yeah

*So in a way it’s positive like as well erm but just having that me and ha getting to be yourself in a way that’s the positive side of it erm and then the when you’re when you’ve come off it you’re positive cause you’ve had it and erm you look forward to the next that’s about it.’*

He mentions that he is glad he gets to have a ‘*totally […] different life’* and that after he comes off the drugs, he returns back to his work life where he remains in control. Azaan feels ‘*glad*’ he has had the experience, otherwise he would be ‘*probably off the rails’* without that release that acted as an affirmation of his MSM identity. Overall, *‘sex on chems’* he felt was ‘*positive*’, a word he used twice for emphasis, as he looks forward to his next release. However, despite Azaan’s apparent literacy with drug management, some might voice an element of concern over the compartmentalisation of his lives and associated self-stigma.

## 4. Discussion

This study shows that the participants in Leicester, Leicestershire, and Rutland are using a range of ‘Chemsex’ drugs, including cocaine, GHB, crystal methamphetamine, ketamine, and mephadrone [51]. This study is the first to show in a non-major UK city that cannabis may also be considered part of the ‘*sex and drugs’* scene, mirroring research with MSM in Canada [52] and Germany [53]. Assessing rates of Chemsex is challenging, but our figures suggest higher participation in sexualised drug use than in other surveys in the United Kingdom and the Republic of Ireland [54]. Responses to the survey indicated that participants enjoyed taking drugs, they mostly knew how to use drugs safely, and that they knew where to go for advice on drugs. Most were also able to enjoy sober sex. However, they also reported that they did things high they would not do sober (including anal sex without PrEP or condoms) and a significant minority struggled to enjoy sex without drugs. The responses to the survey in this study were also similar to research in London, which showed a high risk for STIs and HIV through unsafe sexual practices but also regular engagement with sexual health services and other special services to try to reduce risks [55]. In the survey, the respondents identified they would like support from an LGBT friendly service for ‘Chemsex’; this has been advocated by authors such as Moncrieff [56]. Training LGBT sexual health service providers in harm-reduction techniques would benefit service users and allow for standardized harm-reduction care for GBMSM services. Standardized harm reduction training in GBMSM services should be considered as part of future government policies, with flexibility to allow for localized cultural contexts to emerge and inform the harm-reduction services provided to those who engage in ‘Chemsex’.

Furthermore, while the men in our case study data showed that, for the most part, condoms or anti-HIV medication were being used, the GBMSM who completed the survey self-reported significant incidence of condomless anal intercourse (CAI), mirroring previous research [52]. A study in London and Brighton found CAI occurring, even when drug use during ‘Chemsex’ decreased [57]. The two phases of this study suggests that the findings are somewhat similar to previous research. In phase 1, this study’s survey identified CAI as a potential risk outside of major gay scene cities. In the case studies of phase 2, there were discussions of PrEP (preventing transmission) and ART (undetectable HIV load; therefore untransmittable) to protect against HIV transmission as well as the negotiating of condom use and a high frequency of testing every 3–6 months for STIs, which, as stated, may act to reduce the risks associated.

The other risks that emerged from the case study accounts included risk of overdose death, more common in poly drug users and GHB users [14]. In both the survey and the case studies, polysubstance use was common, which increases the risk of overdose. While health literacy was generally good in all three interview participants, maturity and assumed responsibility in managing a partner’s well-being varied considerably. In his case study, Aabid said he would not call an ambulance but leave the individual out of fear of his MSM identity being exposed with police involvement. This hesitancy to involve law enforcement when ‘Chemsex’ goes wrong is also reported in the literature [58]. In Italy, ways to reduce overdose deaths include writing down in a diary the time when a drug has been used and then trying to space polysubstance [59]. In the case of Angus, he was more informed and had experience with those who had overdosed. However, he took pride in taking care of others and their well-being. Further research should explore experiences of overdose from both the individual who overdoses and those who were around when the overdose occurred, to better understand this within the sex and drugs setting within GBMSM communities. This could include research on a supported mentoring training scheme where those more experienced with overdose share what to do in the event of an overdose to novices.

In the other two case studies, the quality of drugs in LLR was also noted as a risk; this included the illegal import of crystal methamphetamine, with addiction being described as more prevalent in Leicester (though this may be due to the heightened stigma of drug users in a smaller community). Similar to the cases in this study, research with experienced users from Manchester also claimed methamphetamine purity is related to clear crystals, but when the purity was tested, the purity ranged from higher (94%) than Europe (87%) to as low as 22%, showing a variable range that was related to visible clearness. Unlike these cases, where GHB/GHL was also said to vary in purity, the same study in Manchester found GHB/GHL to consistently have high purity [60]. These findings are consistent with previous studies, where quality of drug is self-reported to be a decisional factor in which drugs to choose for a ‘Chemsex’ session [1]; that report, however, differed from this study in that overall drugs were reported to be of lower quality across the board. In the harm reduction literature, users will often avoid buying from a dealer who they perceive provides low quality substances, when in reality, users have little understanding of how to judge the purity of substances without drug-testing kits [61]. This increases the risk of over consumption and overdose, particularly if it is assumed the drug lacks purity as more of the drug is consumed to compensate [62]. This could suggest that services provide either drug-testing kits for purity and cut, or provide service users a list of reliable sources where they can purchase their own testing kits. LLR has a relatively small gay scene, with those in the case studies implying that everyone knows everyone and that confidentiality was a concern when accessing support services. This concern around confidentiality is reported in research outside of cities like London, with men in a small pilot study in Dublin, Ireland, raising concerns of confidentiality [63]. Suggestions to mediate this risk include services having a policy on confidentiality that is clear to service users to encourage uptake of ‘Chemsex’ services [64].

While there were risks, there were also safety measures in place by the participants in the case studies, especially Azaan and Angus. Some of these have also been cited in a recent qualitative synthesis on benefit-enhancing and risk-reducing practices [65]. This included things like meal preparation for after a ‘Chemsex’ session to allow the body to recover. This is the first study to find nutrition management as part of preparation and recovery, and further research is required to better understand eating behaviors within this context. As mentioned, there was frequent STI and BBV testing. This is good practice and should be highlighted, as most research on ‘Chemsex’ does not report how frequently people test for STIs and BBVs. Drugs and paraphernalia were stored safely to keep out of the reach of children, for example, in glass bottles labelled *‘dangerous substance’* in case anyone was to find it. This is another good practice that is missing from the current research and requires further investigation. Crystal methamphetamine was also smoked in a glass pipe, which is considered better than in plastic or metal pipes as, if they are scraped, the individual does not inhale fumes from the metal or plastic. The length of the pipe is also important for preventing the transmission of Hepatitis C from sharing pipes and the burning of lips [66]. Again, this good practice is not currently discussed at length; providing glass pipes with several mouth pieces is another tangible action services can take to reduce BBVs, as well as training service providers on how to provide safer smoking advice such as using sugar to ensure they are not burning off the purity of the drug. There were also use of escorts and negotiation of sexual practices including condom use on Grindr. This area of escorts involved requires further exploration, with both escorts and purchasers of sex. In the case studies, social support was mentioned as a buffer against risks. From previous studies it has been found that GBMSM who have increased social support also have better mental health outcomes, with a call for interventions that combine social support and harm reduction [67]. Finally, the men in the case studies all enjoyed pleasure from ‘Chemsex’ and moderated against several risks, such as taking ART and PrEP to prevent HIV transmission. However, ART adherence has been reported as low in those engaging in ‘Chemsex’ [68], while the reported use of PrEP has been mixed in different studies and has been reported to be high [13,69]. This again is a point for further research with larger samples.

It is important to note that the men in the case studies did not identify with the term ‘Chemsex’ to describe their experience, and in contrast to Stuart [3], the GBMSM in the case studies found the term stigmatising. There was no standard terminology, but services should take time to reflect on what is the appropriate localized and cultural term to use to ensure they reach their target populations. Further localised research is needed to determine the best name to use, with it recommended that services continue to allow the term to emerge from the GBMSM community. Whether terminology in non-binary individuals is different also needs further exploration as in study 1, non-binary individuals outside the queer metropolis are having sex on drugs, and what language they prefer may not be the same as those who identify as cisgender GBMSM.

When considering the qualitative and quantitative findings together, both point to generally good levels of literacy and awareness of support and largely challenge common representations of engaging in ‘sex with chems’ as a maladaptive coping strategy. The case studies provide fuller accounts of both the nature of pleasure and the various rituals and risk-reduction strategies used by the men and consider some of the inter-personal components of these practices. There are some challenges in synthesising the findings as (in our attempt to keep the survey quick to complete and accessible) we were not able to gauge the frequency, location, or duration of sex-on-chems sessions. The three case studies provide in-depth idiographic case studies which may be useful for training purposes and offer original cultural insights, especially around stigma, gay identity, and ‘Chemsex’ for men from the British Muslim community. However, because of both the number and nature of the case studies, the authors do not claim transferability of the qualitative findings which is typical of the interpretative phenomenological approach.

The findings from this study are in line with the Normalisation Theory where most users did not generally see their sexualised poly drug use as problematic in either the survey or case studies. There has been increasing evidence that drugs such as amphetamines have become an accepted part of everyday culture under the theory, which was originally developed from 800 British drug-using youth. In the last 20 years, the theory has been tested in other high-income countries such as Europe, Australia, the United States of America, and New Zealand, with attention paid to how normalisation informs practice and experiences of drug use [70]. The theory has been extended to explain the normalisation of drug use in the gay community, particularly in the Australian gay community, which suggests there is little point of promoting that people should abstain from drugs as they are a normal part of the gay scene. These findings emerged in the mid-1990s and were confirmed in interviews with gay men who did and did not use drugs [71]. So called ‘party drugs’, which include amphetamines and ecstasy, were reported as often being combined with alcohol or other drugs, resulting in poly drug use being normalised in the gay community, similar to participants in this study. In general, there are further micro-politics at play within the normalisation theory, with neo-liberal political discourses playing on the stereotype of an excessive drug user in an attempt to try to control and stifle pleasure for some individuals who do not want to regulate their substance use, such as in the case studies. In LLR, the local government budget cuts and lack of evaluation of sexual health strategies result in a political climate where the right to pleasure for GBMSM may appear as a priority on one strategy and then disappear on the next, with no evaluation to provide evidence if the strategy was effective and what it achieved, leading to inconsistent and insecure support. Challenging the neo-liberal politics of the Normalisation Theory though, GBMSM in LLR who engage in sex on chems can benefit from the LGBT+ sexual health service and local drug service’s Chemsex clinic drop-in, even if the local political and financial climate change, though it has to be said the charity sector in England is also struggling in the current financial climate. Regardless, these services are independent and can advocate on behalf of GBMSM to ensure stereotypes do not remain unchallenged and that the needs of the community they serve are heard. Furthermore, they can work with researchers to collect data to evidence the need for a local service to remain from one sexual health strategy to the next. Particularly, as we live in a time of individuality, ‘sex on chems’ allows for a collective behaviour of pleasure in the social world, creating a sense of belonging to a group engaging in subcultural practices that go against the hegemonic norm of neoliberalism [72]. One London study found that gay men consider it normal to have a sex party environment where gay men who are HIV positive can inject drugs and have CAI, without restrictions on pleasure. The HIV-positive men reported that they have nothing left to lose and they cannot contract HIV again so are not motivated to restrict their pleasure [25]. In this study, Angus’ account in particular can be understood through the lens of normalisation theory, with the individual taking responsibility to use ART to control their HIV viral load to be undetectable and untransmittable. However, in LLR, 42.1% of residents are migrants, making it important to note that in the local context some GBMSM may not have had the same access or felt able to access HIV testing or PrEP in their native country. It is important that new arrivals have access to sexual health information such as where to go for HIV and STI testing in their native language from trusted LGBT+ services and that this is provided for all new arrivals. This leaves several questions for those providing harm reduction, such as how to work with individuals who do not want their pleasure regulated, and requires further exploration into how harm-reduction advice can be provided in a way where the GBMSM micro-politics and normalisation theory account for how pleasure and harm reduction can co-exist [73]. This is why a holistic model combining localized intel from GBMSM sexual health services and harm reduction could be a fruitful model to explore in further research.

The Minority Stress Theory frames the psychological well-being of the participants in phase 1 and phase 2. A systematic review on mental health and ‘Chemsex’, found mixed findings on whether there is a link between Minority Stress Theory and drug abuse on the scene [74], with stigma becoming a barrier to seeking information or accessing harm reduction services, as well as acting as a barrier to counselling [56]. Phase 2 of this study suggested some stigma may exist when seeking support for well-being. However, this study also showed that identity processing conflict was low and was considered to be processed and resolved in their youth. As adults, they were managing their identities in various ways that worked for them without stigma. In the case studies, discussions were had around access to confidential support, particularly for MSM individuals who could face real or perceived harm, resulting in poor well-being, from those who stigmatize their sexual identity. This is a particularly pertinent concern in LLR, given that homosexuality is stigmatized in the main countries that residents migrate from [75,76,77,78]. Future research should continue to tease out the nuances of the aspects of Minority Stress Theory that do impact on well-being, recognizing differences amongst the GBMSM group, and that not all proximal and distal stressors will present. Support for the theory of Wild Self-care can also be seen in the case study accounts, with nutrition to replenish the loss of serotonin and stress release emerging, and this could be further considered to balance the minority stress identified with experiences of pleasure to obtain a holistic understanding of well-being during sex on chems.

Several suggestions of tangible actions for services who work with GBMSM who engage in ‘sex on chems’ have been provided above, with attention being paid to core harm-reduction techniques that are transferable to specialized GBMSM sexual health services, ensuring that services do not seek to police bodily pleasure, and emphasising the need for standardized education and policy recommendations that aim to be flexible enough to be localized. For example, LLR saunas for GBMSM could provide support, or online drop-in clinics specifically for rural residents in LLR (though the LGBT+ sexual health charity does outreach on a popular gay hook-up app).

### Limitations and Future Research Directions

There are limitations to this study that are important to discuss. First, the study included a small sample of case studies and the authors do not claim that these findings would transfer to other members of the local GBMSM community. Indeed, the three accounts diverge in interesting ways showing the complexity of sexualised drug-use accounts. Additionally, all interviews were one-off and there was no follow-up to see how practices may change over time. Longitudinal studies are needed on GBMSM who engage in ‘sex on chems’ outside of the queer metropolis. Second, a power calculation for representation in the pilot survey could not be estimated; the ONS and other institutions that collect data on sexual orientation need to be increased in rigor by separating gay from lesbian responses, provide a way to capture MSM responses, and separate male from female bisexual responses. Third, the study survey was a pilot and lacked comprehensive validation and we could have captured additional data or used a comparison to non-drug users. If there were more respondents, a meaningful conversation around ethnicity and culturally appropriate resources could have been made. While harm prevention and risk reduction are understandably pivotal within the current research and practice, to achieve a more nuanced and holistic understanding of this subcultural behavioural phenomena, there needs to be understanding in a more sophisticated and integrated way [79]. Some recent scholars have introduced the term ‘critical Chemsex studies’ to explore the interplay of public health and how intimacy, identity, and community equate with both the meanings and experience of homoerotic pleasure, and to interrogate the ‘discursive, socio-political, technological and economic landscapes in which ‘Chemsex’ has materialised’ [80]. Our findings capture some of these elements in passing, providing further evidence of some of the multiple meanings and motives for ‘sex on chems’. For the purpose of this analysis, we have focused on understandings and experiences of risk management primarily but there is potential to develop these under-researched aspects more fully in the future, and in particular, we recognise that in the quantitative component, potentially, we could have explored participants’ motivations and explanations for engaging (or not) in sex on chems. For various logistical reasons, unfortunately, it has taken some considerable time to collate both sets of data and write up for publication. As we have acknowledged, the ‘sex on chems’ scene is highly fluid and dynamic and practices and substances will have evolved since our data were collected. Other contextual factors, such as increased availability and usage of PrEP by British GBMSM [81] and reductions in sexual health and substance-use agencies, have been acknowledged above. Nonetheless, we believe our findings illuminate some of the complexities of ‘sex on chems’ perspectives and practices in a unique social-cultural context and offer a unique contribution to this salient and stigmatised field of public health.

## 5. Conclusions

In conclusion, while there appears to be some similarities between the queer metropolis and this study, in this study, a special case has been made to consider how different the experiences are of GBMSM in Leicester, Leicestershire, and Rutland, which has a small gay scene compared with other cities like London, Brighton, and Manchester. Further research is needed outside of the queer metropolis, with particular focus on the different nuances of local cultural practices in order to tailor harm reduction and education-based interventions. These nuances are important for commissioners and policy makers to ensure the needs of GBMSM who have ‘sex on chems’ outside large gay scenes are met. Looking forward, in this fast-moving area of sexual and mental health research and practice, innovation is key. As we further expand, critical chemsex studies’ interdisciplinarity and the integration of competing discourses are important. There are multiple and sometimes shifting motivations for engaging in sex on chems and the associated emotions experienced. Research methods that facilitate expressive arts-based ways of capturing these complex emotional and embodied experiences and/or the use diary/voice-note methods that can capture accounts of an experience closer to the event may be beneficial and have thus far been little utilised in this field. The development, evaluation, and sharing of good quality, accurate, stigma-reducing resources, potentially co-created with experienced sex-on-chems participants, needs to be another priority for agencies with psychoeducation on the effects of various substances alongside clear guidance on behavioural strategies to minimise risk, ensure consent practices and processes, and encourage the collective responsibility of participants in encounters.

## Figures and Tables

**Table 1:** Attitudes Around ‘Chemsex’.

Questionnaire Item	Strongly Agree	Agree	Neither Agree Nor Disagree	Disagree	Strongly Disagree
*I enjoy taking drugs*	47%	29%	12%	6%	6%
*I know how to use drugs in a safe way*	57%	27%	12%	4%	0%
*I feel like my drug use is having a negative effect on my life*	35%	12%	16%	23%	14%
*When I’m high/on drugs I do things sexually that I wouldn’t do sober*	47%	37%	8%	6%	2%
*I’m more likely to have unsafe sex when I’m high/on drugs*	31%	45%	12%	6%	6%
*I am able to enjoy sex without using drugs*	43%	23%	22%	12%	0%
*If I wanted advice about my drug use I would know where to go*	41%	33%	4%	16%	6%

## Data Availability

Data are available upon reasonable request due to the sensitive nature.
